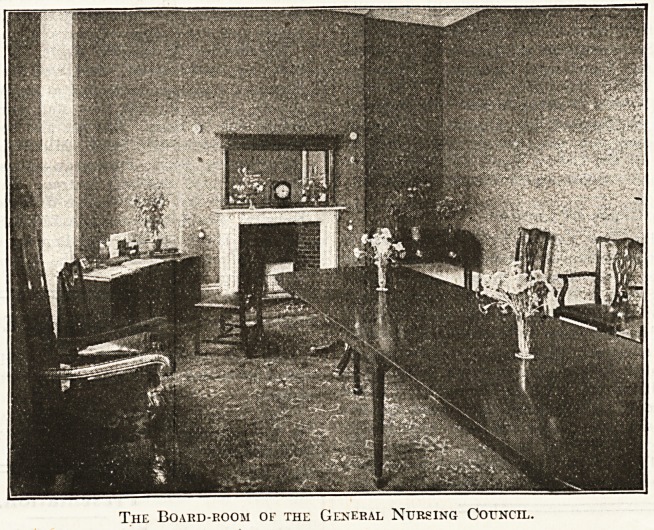# Opening of the Official Home

**Published:** 1921-06-18

**Authors:** 


### THE NURSING REGISTER OF ENGLAND AND WALES.

### Opening of the Official Home.

FRIDAY, June 10, was a great day in the annals of the General Nursing Council for England and Wales. The Council has carried on its work since May 1920, when it was constituted under the Act of 1919 in cramped quarters which were the best that the Ministry of Health could grant it. On Friday the headquarters at 12 York Gate, Regent's Park, a well-built and commodious house at the corner of Marylebone Road, facing Marylebone Church, where the Council had moved two days previously, were opened by H.R.H. Princess Christian. Despite the shortness of the invitation, a number of guests assembled for the opening, amongst whom were: The Countess of Kenmare, Lady Morant, Mr. and Mrs. L. Brock, Mrs. Priestley, Miss Beadsmore Smith, R.R.C. (matron-in-chief, Q.A.I.M.N.S.), Miss Cruickshank (matron-in-chief, Royal Air Force Nursing Service), the Venerable the Archdeacon of London, the Hon. Sir Arthur Stanley, G.B.E., the Mayor of Marylebone, Canon Sprankling, Miss Dora Finch (University College Hospital), Miss M. E. Rundle, R.R.C. (secretary of the College of Nursing), Miss McIntosh, C.B.E., R.R.C. (matron, St. Bartholomew's Hospital), Miss Hogg, C.B.E. (matron, Guy's Hospital), Miss Barton, R.R.C. (matron, Chelsea Infirmary), Miss Beatrice Kent, Miss Ellen Pearce, Mrs. Paul, Miss Clark (matron, Whipps Cross Hospital), Miss Dodds (matron, Bethnal Green Infirmary), Major R. W. Barnett, M.P., Miss Hare, Mr. Alfred Langton, Mr. Cavendish Bentinck, together with Mr. J. F. Priestley, K.C. (chairman of the General Nursing Council) and the members of the Council.

The opening ceremony took place in the Board Room on the first floor, where Princess Christian was conducted on her arrival by Mr. Priestley, Mrs. Bedford Fenwick, and other members of the Council, with whom was the Archdeacon of London. In welcoming the Princess, Mr. Priestley referred to Her Royal Highness's great interest in the cause of Registration and to the work of those through whose efforts the seed sown by Mrs. Bedford Fenwick and her friends in 1887 had been brought to fruition—viz., Miss Isla Stewart, Miss Louise Stevenson, Mrs. Kildare Treacy, Sir Victor Horsley, and the late Sir Robert Morant (all of whom have passed away), Dr. Bedford Fenwick, Dr. Farquharson, who introduced the first Bill into Parliament in 1904, which was followed by that of Lord Novar (then Mr. Munro-Ferguson) in 1905; the following societies: The British Medical Association, the Royal British Nurses' Association; the Matrons' Council for Great Britain and Ireland, the Society for the State Registration of Trained Nurses, the Fever Nurses' Association, the Irish Nurses' Association, the Scottish Nurses' Association, the College of Nursing; Lord Ampthill, Captain Barnett, Dr. Chapple, Dr. Addison (the late Minister of Health, who made the Bill a Government measure), and his assistant secretary, Mr. L. V. Brock. Of his colleagues on the Council Mr. Priestley said they were well chosen and pleasant to work with. The delay in opening the English Register, which was ready last October, has been due to the necessity to consult with Scotland and Ireland, but now the Rules for existing nurses have been finally passed by the Council and have been sent to the Minister for approval and signature under the Act. Mr. Priestley, in his concluding words, indicated the work which will in future be carried on in the Council's premises for the education and examination of nurses, for the affiliation of smaller hospitals and infirmaries to the larger schools of training, and for all that will tend to advance the interests of the nursing profession. The Venerable the Archdeacon of London then invoked God's blessing, praying that the members of the General Nursing Council may be granted clearness of vision, guidance in their deliberations that diversity of opinions may lead to unity in decisions, tact with themselves, with each other, and those with whom they are acting, power to organise, love in organising and courage in their work. He dedicated the house to God "in thanksgiving for all that has been done and all it stands for."

THE BOARD-ROOM OF THE GENERAL NURSING COUNCIL.

Her Royal Highness then said: "It gives me the greatest pleasure to declare these new premises of the General Nursing Council to be open, and personally to congratulate you on the recognition and protection which the Registration Act gives to your profession. Your Council is representative of all parties in that profession, and I know that all will join hands in one common endeavour to promote the well-being and prosperity of the nursing profession, so that this house may become a temple of harmony and peace from which will emanate great and beneficent influence for the relief of suffering and the preservation of health."

Presentations to Princess Christian were then made, while the general company dispersed for tea and an inspection of the premises. The task allotted to the sub-committee, consisting of Mrs. Bedford Fenwick, Miss Cox-Davies, and Miss Villiers, with the Registrar, of decorating and furnishing for the purposes of the Council's headquarters the house which has been leased from the Bedford College trustees, cannot fail to have been an exceedingly pleasant and interesting one, and the result shows that the amount of personal interest and pains bestowed on the work must have been considerable. If beauty is the perfection of function, then beautiful may be the word justly ascribed to the furniture and fittings at 12 York Gate. On the ground floor are the waiting-room, the Assistant Registrar's room, and the registration-room, with a small cloak-room and lavatory; on the first floor are the Board-room and the Registrar's room; on the second floor are three rooms, two of which are for the accountant and for stencilling work, and a lavatory and bathroom; and on the top floor is a self-contained flat of four rooms, two bedrooms, kitchen, and sitting-room, for the housekeeper and her assistant; in the basement is an excellent tea-room for the Registrar's staff.

## Figures and Tables

**Figure f1:**